# An Enzybiotic Cocktail Effectively Disrupts Preformed Dual Biofilm of *Staphylococcus aureus* and *Enterococcus faecalis*

**DOI:** 10.3390/ph16040564

**Published:** 2023-04-10

**Authors:** Salim Manoharadas, Naushad Ahmad, Mohammad Altaf, Abdulwahed Fahad Alrefaei, Basel F. Al-Rayes

**Affiliations:** 1 Department of Botany and Microbiology, College of Science, King Saud University, P.O. Box 2454, Riyadh 11451, Saudi Arabia; 2 Central Laboratory RM 63AA, College of Science, King Saud University, P.O. Box 2454, Riyadh 11451, Saudi Arabia; anaushad@ksu.edu.sa (N.A.); maltaf@ksu.edu.sa (M.A.); bfalrayes@ksu.edu.sa (B.F.A.-R.); 3 Department of Chemistry, College of Science, King Saud University, P.O. Box 2454, Riyadh 11451, Saudi Arabia; 4 Department of Zoology, College of Science, King Saud University, P.O. Box 2454, Riyadh 11451, Saudi Arabia; afrefaei@ksu.edu.sa

**Keywords:** enzybiotics, biofilm, antimicrobials, *Staphylococcus aureus*, *Enterococcus faecalis*, diabetic foot ulcers

## Abstract

Multidrug-resistant bacterial infections are on the rise around the world. Chronic infections caused by these pathogens through biofilm mediation often complicate the situation. In natural settings, biofilms are often formed with different species of bacteria existing synergistically or antagonistically. Biofilms on diabetic foot ulcers are formed predominantly by two opportunistic pathogens, *Staphylococcus aureus* and *Enterococcus faecalis*. Bacteriophages and phage-based proteins, including endolysins, have been found to be active against biofilms. In this study, we evaluated the activity of two engineered enzybiotics either by themselves or as a combination against a dual biofilm formed by *S. aureus* and *E. faecalis* in an inert glass surface. An additive effect in rapidly disrupting the preformed dual biofilm was observed with the cocktail of proteins, in comparison with mono treatment. The cocktail-treated biofilms were dispersed by more than 90% within 3 h of treatment. Apart from biofilm disruption, bacterial cells embedded in the biofilm matrix were also effectively reduced by more than 90% within 3 h of treatment. This is the first instance where a cocktail of engineered enzybiotics has been effectively used to impede the structural integrity of a dual biofilm.

## 1. Introduction

The extracellular polymeric substances (EPS) formed by biofilms provide a safe niche for the sessile microbial community. The biofilms-embedded bacterial life is one among the predominant forms that exist in nature. In comparison to the free-living or planktonic mode of life, biofilm-embedded cells exhibit a completely different behavioral pattern [1]. Clinically relevant biofilm-mediated chronic infections are difficult to treat, primarily due to the inability of the antibiotics to reach the biofilm-entrapped bacterial cells. In addition, biofilms also confer protection to bacteria against several physiological and chemical stresses, predation, and mechanical forces [2,3,4,5]. Biofilms that occur in nature are frequently comprised of various species of bacteria, which may interact with each other antagonistically, commensally, or synergistically [6]. The interactions between bacterial species in a biofilm-associated community vary temporally with the microenvironmental conditions such as oxygen level, nutrient availability, temperature, and quorum-sensing signals [7].

The opportunistic pathogens *S. aureus* and *E. faecalis* are leading causes of nosocomial infections [8,9]. Both pathogens can cause infections mediated by biofilm formation such as UTI (Urinary Tract Infections), wounds, and endocarditis [10,11,12,13,14,15]. Polymixed biofilms are often caused by *S. aureus* along with *Enterococcus* sps., *S. pyogenes*, *P. aeruginosa*, and *C. albicans* [16,17,18,19,20]. These multimicrobial biofilms are hard to eradicate and cause chronic and persistent infections, ultimately contributing to a high mortality rate [21]. According to the antimicrobial surveillance program (SENTRY: JMIlabs), *S. aureus* contributes to 45.9% and *Enterococcus* sp. 8.2% of skin and soft tissue infections [22]. Skin and soft tissue abscesses have fluid and pus-filled chambers often infiltrated by inflammatory cells and bacteria and are resistant to the treatment with conventional antibiotics [23]. Around 20% of diabetic patients suffer from diabetic foot ulcers during their lifetime [24]. Interestingly, polymixed biofilm colonization in diabetic foot ulcers is prevalent with the commonly associated pathogen being *S. aureus* (54.8%), followed by *E. faecalis* (45.2%) [25]. In addition, the attachment and biofilm formation of *S. aureus*, *E. faecalis*, and *S. epidermidis*, on the surface of medical implants and instruments, is another common cause of bacteria-associated nosocomial infections [26].

It has been experimentally proved that bacteriophages can disrupt bacterial biofilms. This is typically achieved by encoding several enzymes such as lysins and depolymerases [27]. There have been reports of 160 putative depolymerase enzymes from 143 different bacteriophages [28]. These depolymerase enzymes can be broadly classified as hydrolases and lyases [28]. Apart from intact phages, phage-derived proteins, such as lysins, have been shown to be efficient in the removal of preformed biofilms. For instance, *Staphylococcus* lysin P128 was found to be active against Methicillin-sensitive *Staphylococcus aureus* (MSSA) and Methicillin-resistant *Staphylococcus aureus* (MRSA) strains [29]. In a similar study, an engineered phage-based lysin, LysAB2, was shown to be active against *Acinetobacter baumannii* and could disrupt its biofilm formed on a 96-well plate at the end of 24 h [30]. Interestingly, it was also shown that these phage-based proteins could be used in combination with antibiotics, nanoparticles, and other endolysins [31,32]. A synergistic action was seen with poly-N-acetylglucosamine depolymerase DA7 with LysK endolysin in degrading and disrupting *S. aureus* biofilms, both in dynamic and static models of infection [33].

Our earlier studies have shown that an engineered lysin, BP404, was active against *E. faecalis* [34]. In this study, we evaluated the activity of BP404 combined with another engineered endolysin, P16-17/100, against a mixed biofilm formed by a veterinary isolate of *S. aureus* and a clinical *E. faecalis* isolate. Both the proteins were efficient in removing the biofilms and targeting the biofilm-encased bacteria, with the enzybiotic cocktail proving to be extremely efficient in biofilm disruption and killing biofilm-associated bacteria. This is one among the very few studies in which a cocktail of endolysins has been evaluated for efficiency against dual bacterial biofilm.

## 2. Results

### 2.1. Construction, Synthesis, and Purification of Protein P16-17/100

**Figure 1:**
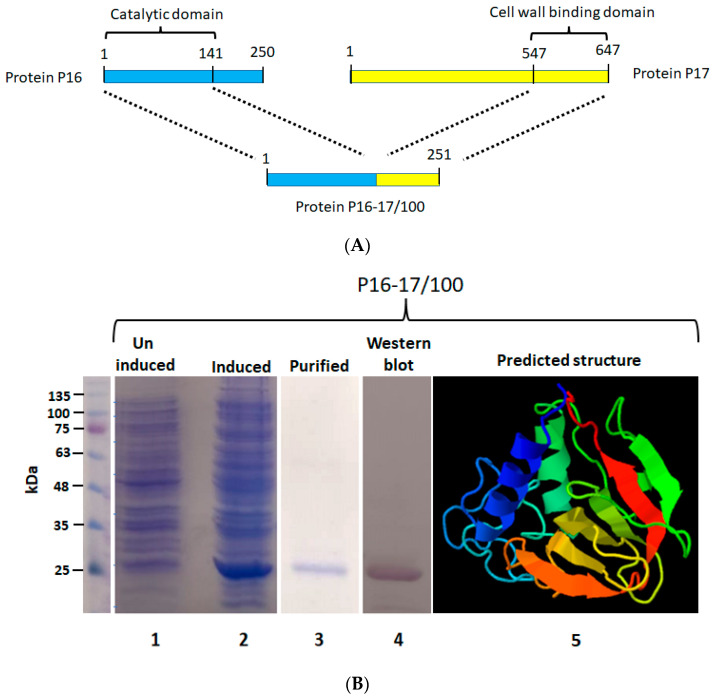
Construction of protein P16-17/100. (**A**) The catalytic domain from protein P16 (141 aa) was linked to the cell wall binding domain of protein P17 (100 aa). (**B**) Expression and purification of protein P16-17/100. Lane 1 shows the uninduced culture without synthesis of the protein. The induced culture showing synthesis of protein P16-17/100 is shown in lane 2. The Ni-NTA-purified protein is shown in lane 3. The Western blot performed to detect the protein is shown in lane 4. The structure of P16-17 predicted by the Phyre program is shown in lane 5. Violet to Red (rainbow colors) change represents N-terminal to C-terminal domain of the protein.

The chimeric protein P16-17/100 was constructed by linking 141 amino acids (catalytic domain) from the endolysin P16 of the bacteriophage ϕ44AHJD with a 100-amino-acid cell wall binding domain from the minor tail protein P17 of phage ϕ44AHJD (Figure 1A). The protein P16-17/100 was produced by IPTG-mediated induction of the gene P16-17/100 cloned under a lacUV5 promoter in the expression vector pQE30. After induction with IPTG, the expression was carried out at 16 °C. The protein synthesis was confirmed by resolving the uninduced and induced samples on a 12% denaturing PAGE gel. As shown in Figure 1B (Lane 2), the protein synthesis was seen in the induced samples and was absent in the uninduced samples (Figure 1B: Lane 1). The molecular weight of P16-17/100 was judged to be 27 kDa. The protein was found to be expressed in inclusion bodies, so denaturation purification was done. Ni-NTA chromatography was employed to purify the expressed proteins. The purified protein was checked for its purity by resolving onto a 12% PAGE gel. The protein was visually judged to be more than 95% pure from the gel (Figure 1B: Lane 3). The purity of the protein was further assessed by Western blot analysis with antibody directed against the 6× histidine tag at the N-terminal region of protein P16-17/100. As seen in Figure 1B: Lane 4, only a single band corresponding to the expected protein size (27 kDa) was seen with no degradation products. The predicted three-dimensional structure of P16-17/100 by the Phyre2 program [35] is shown in Figure 1B: Lane 5. The purified P16-17/100 protein was assessed for antibacterial activity against the *S. aureus* Rumba strain.

### 2.2. P16-P17/100 and BP404 Displays Activity against Planktonic S. aureus Rumba

**Figure 2:**
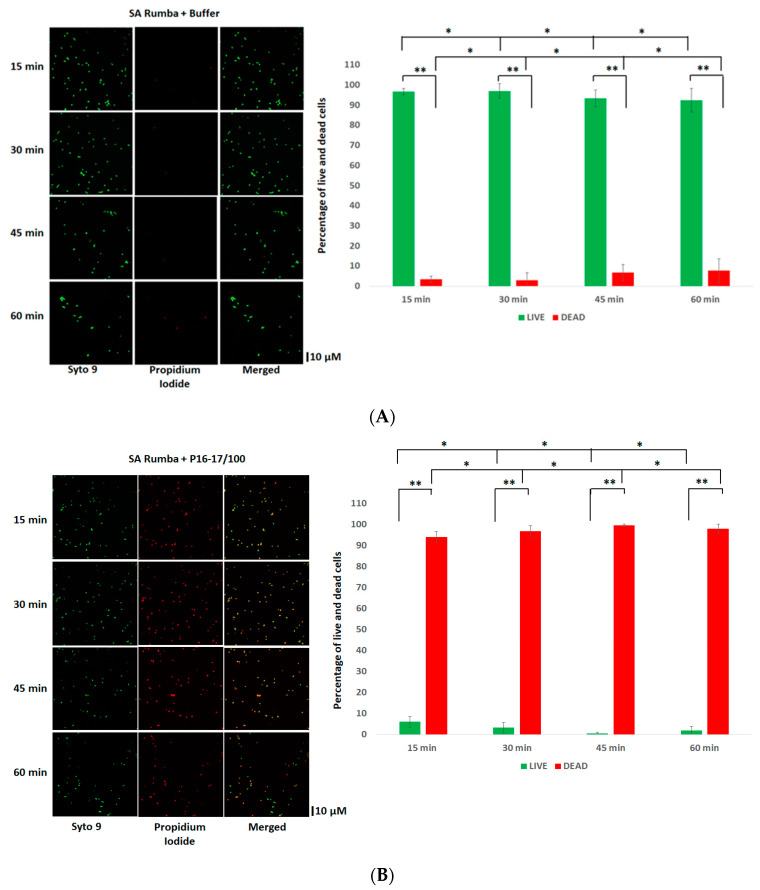

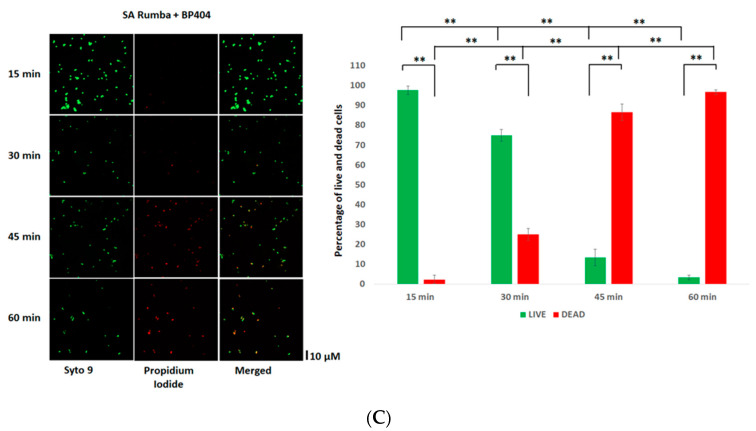
Activity testing of P16-17/100 and BP404 against *S. aureus* Rumba planktonic cells (SA) Rumba. (**A**) SA cells were treated with buffer, and live and dead cells were estimated at various time points. The Syto9 channel shows live cells, and the propidium iodide channel shows dead cells. The merged channel shows both live and dead cells. The percentage of live and dead cells is shown as a graph. (**B**) Microscopic images of P16-17/100-treated SA cells at various time points is shown. The corresponding percentage of live and dead cells is represented as a graph. (**C**) SA cells treated with BP404 at various time points are shown. The corresponding graph showing live and dead cells is represented as a graph. The error bars show the standard deviation. The statistical test of variance was calculated by one-way ANOVA (* *p* ≥ 0.05; ** *p* ≤ 0.05). The experiment was performed in triplicate.

Initially, we wanted to test the efficiency of both peptides, P16-P17/100 and BP404, against planktonic *S. aureus* Rumba. Logarithmically growing *S. aureus* Rumba cells (1 × 10^7^ CFU) were exposed to 10 µg/mL of both purified proteins. The cell death after protein addition was monitored over a period of 60 min at 37 °C. As seen in Figure 2A, no significant death of cells was seen in the buffer-treated control, with more than 90% of cells alive (green) even after 60 min of treatment. The ratio of live to dead cells is represented as a graph. In contrast, protein P16-17/100 was significantly active against *S. aureus* Rumba cells (Figure 2B), with more than 90% of cells dead within 15 min after addition of the protein. Surprisingly, BP404 was also active against *S. aureus* Rumba cells, albeit at a lower efficiency in comparison with protein P16-17/100. Although there was no significant number of dead cells within 15 min of treatment, the number of dead cells increased with a prolonged incubation, with 20% dead cells within 30 min of treatment. The percentage of dead cells further increased to more than 90% within 60 min following the treatment with BP404 (Figure 2C).

BP404 is comprised of a catalytic domain at the N-terminal targeting a critical peptidoglycan bond. However, the C-terminal was composed of a cell-wall-targeting domain from the phage ϕ1 endolysin PlyV12 [34], expected to bind to *E. faecalis* strains. This observation of the activity of BP404 against *S. aureus* Rumba was surprising. We wanted to confirm the cell-wall binding efficiency of both the chimeric proteins against both *S. aureus* Rumba and *E. faecalis*.

### 2.3. P16-17/100 and BP404 Displays Cell-Wall Binding towards S. aureus Rumba and E. faecalis Clinical Isolate

**Figure 3:**
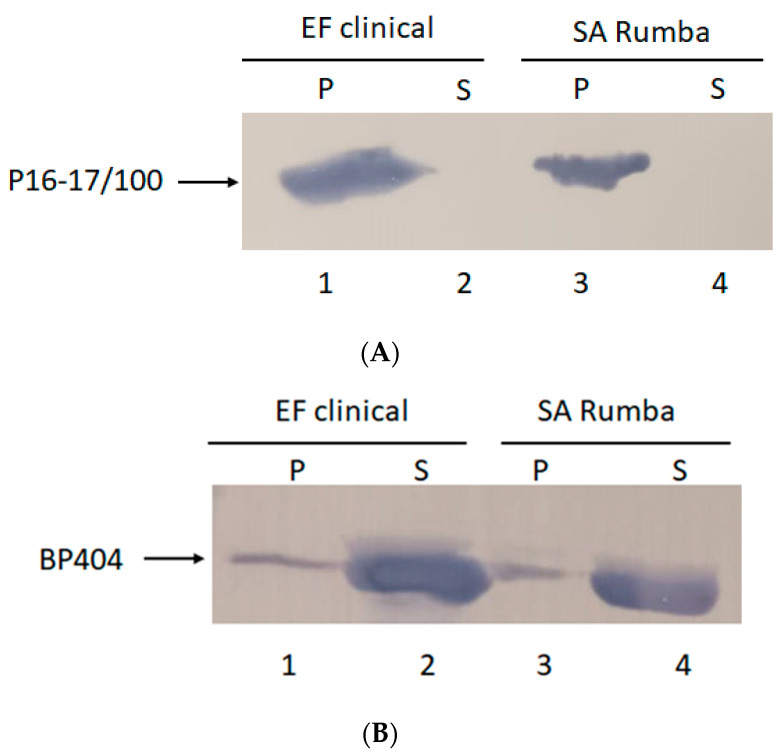
Cell-wall binding assay to determine the binding of protein P16-17/100 and BP404 towards clinical *E. faecalis* clinical isolate and *S. aureus* Rumba. (**A**) P16-17/100 protein was able to bind to the cells of *E. faecalis* (lane 1) and *S. aureus* Rumba (lane 3). No unbound protein was found in the supernatant fraction (lane 2 and lane 4). (**B**) Partial binding of BP404 was found towards *E. faecalis* clinical (lane 1) and *S. aureus* Rumba (lane 3). Most of the protein was found in the unbound supernatant fraction (lane 2 and lane 4).

Protein BP404 was able to display activity against *S. aureus* Rumba planktonic cells. We wanted to confirm by cell-wall binding assay if BP404 was able to bind to *S. aureus* Rumba cells. In addition, we also wanted to test if protein P16-17/100 was able to bind to *S. aureus* Rumba and *E. faecalis* clinical isolate. It was earlier shown that BP404 was able to bind to *E. faecalis* clinical isolate, but not very efficiently [34]. As shown in Figure 3A, protein P16-17/100 could efficiently bind to both *E. faecalis* clinical isolate (Figure 3A: Lane 1) and *S. aureus* Rumba (Figure 3A: Lane 3). Almost 100% of the protein was bound to the cell pellet and no protein was seen in the unbound supernatant fraction (Figure 3A: Lane 2 and Lane 4). In corroboration with a previously observed result described by Manoharadas et al. [34], BP404 was able to bind to the *E. faecalis* clinical strain at a much lower rate, with only approximately 20% of the protein bound to the cell pellet (Figure 3B: Lane 1). Similarly, BP404 was also able to bind to *S. aureus* Rumba, but at a very low intensity (approximately 15–20%, Figure 3B: Lane 3). Majority of the protein BP404 was found in the unbound supernatant fraction. However, this bound amount of protein was enough to display a strong antibacterial activity, as shown in Figure 2.

### 2.4. Formation of Mono or Mixed Biofilm in Inert Surface

**Figure 4:**
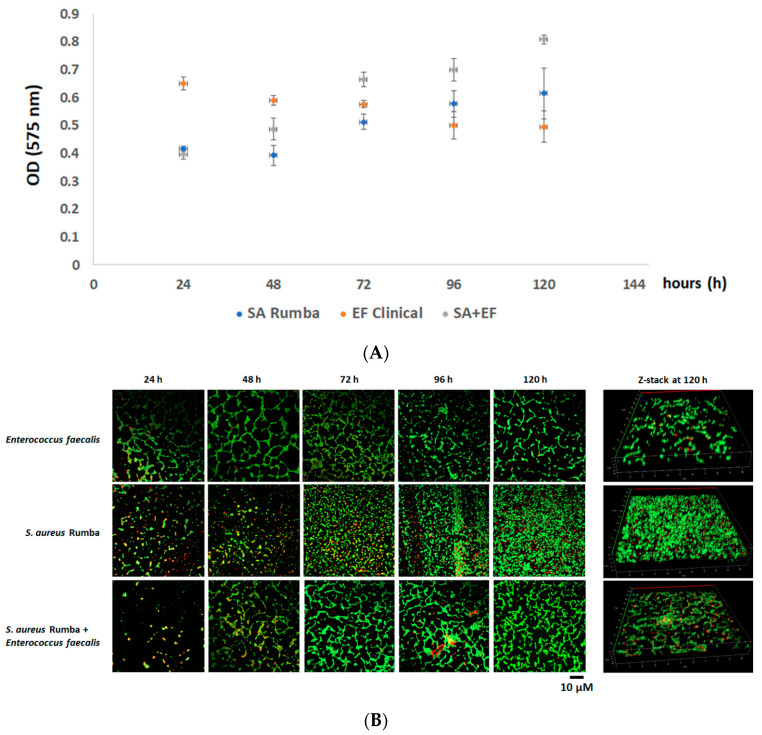
The formation of biofilm by *E. faecalis* clinical isolate and *S. aureus* Rumba. (**A**) Crystal violet staining to measure the thickness of the mono and dual biofilms formed by *E. faecalis* clinical isolate and *S. aureus* Rumba. The thickest biofilm was formed by the dual biofilm at the end of 120 h of growth. (**B**) Microscopy image of biofilm-embedded cells. Green shows live cells and red shows dead cells. The three-dimensional image of biofilm at the end of 120 h is shown as z-stack. The experiment was performed in triplicate, and error bars show the standard deviation.

The efficiency of *S. aureus* Rumba and *E. faecalis* clinical isolate in forming biofilm on an untreated glass surface was evaluated. It is difficult to form bacterial biofilms on a smooth polished glass surface, especially due to the hydrophilic nature of the surface [36]. Our previous studies have shown that *S. aureus* Rumba was able to form strong biofilm on glass surfaces [37]. However, it was not evident if *E. faecalis* clinical isolate was able to form a robust biofilm on glass surfaces. In addition, we wanted to test the ability of the formation of a dual biofilm community formed with *S. aureus* Rumba and *E. faecalis* clinical isolate. Sterile glass coverslips were immersed in LB media, and the biofilm formation was evaluated at designated time points starting from 24 h till 120 h after initial inoculation. Crystal violet staining was also done to detect the robustness and thickness of the biofilm. As shown in Figure 4A, *S. aureus* Rumba formed strong biofilm by 96 h. The *E. faecalis* clinical isolate formed a healthy biofilm within 24 h of growth. However, no increase in biofilm thickness was evident in the further evaluated time points (Figure 4A). The most robust biofilm was noticed in the dual biofilm section with absorbance (OD_575_) value peaking to 0.8, at the end of 120 h, which was higher than both mono biofilm-forming sections. A confocal microscopy was done to visually analyze the biofilm community and particularly the ratio of live and dead cells in the biofilm. As seen in Figure 4B, intact matt-like cell growth was seen in *S. aureus* Rumba biofilm followed by the dual biofilm at the end of 120 h of growth. The 3-dimensional structure of the biofilm at the end of 120 h is shown as z-stack (Figure 4B).

### 2.5. Dual Biofilm Was Effectively Disrupted by a Cocktail of P16-17/100 and BP404

**Figure 5:**
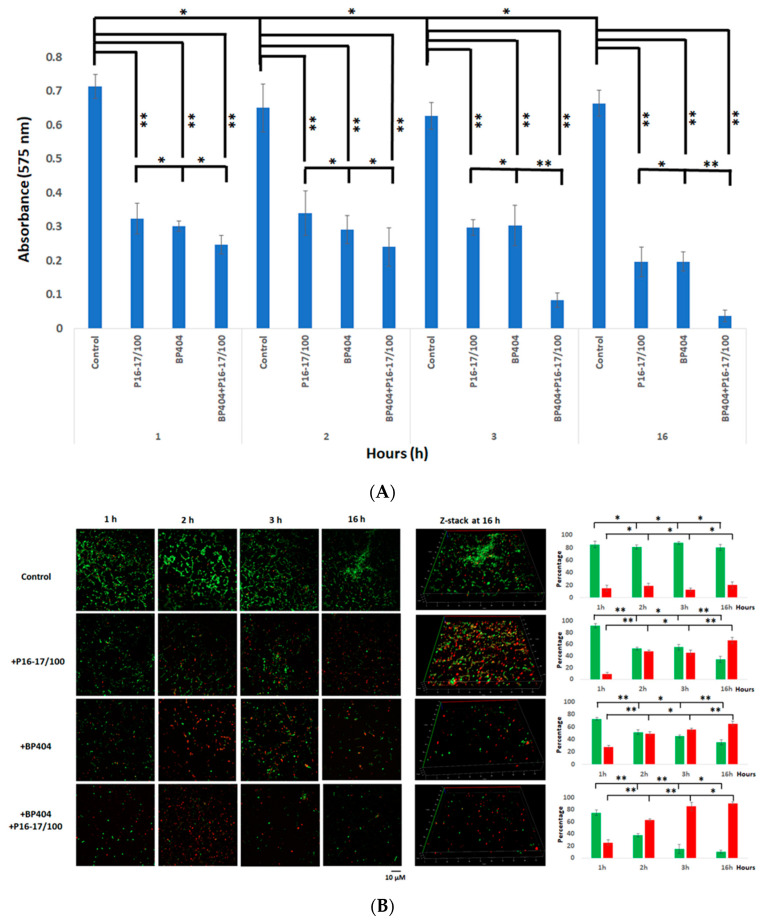
Disruption of biofilm and intensity of dead cells in protein/buffer-treated samples. (**A**) Crystal violet staining of the control and protein-treated biofilm is shown as a graph. The best biofilm disruption was observed with the cocktail of BP404 and P16-17/100 against dual biofilm at the end of 3 h and 16 h following treatment. (**B**) Microscopy images of pre-formed dual species biofilm treated with buffer/proteins at specific time points. The 3-dimentional images following treatment at the end of 16 h are shown as z-stack. The indicative percentage of live and dead cells is plotted as a graph (green: live cells; red: dead cells). The experiment was performed in triplicate. Error bars represent standard deviation. The test of variance was calculated by one-way ANOVA (* *p* ≥ 0.05; ** *p* ≤ 0.05).

In this experiment, we analyzed the effectiveness of individual or a combination of proteins, P16-17/100 and BP404, in disrupting a dual biofilm formed by *S. aureus* Rumba and *E. faecalis* clinical isolate. The treatment of the dual biofilm with a combination of P16-17/100 and BP404 (5 µg/mL + 5 µg/mL) was very efficient in the disruption of the biofilm. A notable effect was seen at 3 h following the treatment where the absorbance at OD_575_ values dropped to less than 0.1 in comparison with the buffer-treated control, which was 0.7 at 3 h (Figure 5A). A further reduction was observed by 16 h after the treatment. Similarly, the individual protein (10 µg/mL)-treated biofilm samples also witnessed a reduction in intensity to 0.3 within 1 h. The biofilm intensity stayed the same until 3 h of evaluation (Figure 5A). A further decline in the biofilm intensity to 0.2 was observed within 16 h of incubation. We further monitored the reduction in live cells encased in the biofilm matrix by microscopy. The dual biofilm treated with P16-17/100 caused a reduction in live cells that amounted to only 50% by the end of 3 h after treatment (Figure 5B). The rate of decline in the number of live cells increased to 60% following further incubation to 16 h. However, BP404-treated biofilm had a decrease in live cells to 60% within 3 h of treatment, with the values rising to 70% with further incubation until 16 h of treatment. When the dual biofilm was treated with a cocktail of P16-17/100 and BP404, an additive effect of reducing the live cells to >80% was noticed within 3 h of treatment, which further increased to 90% within 16 h of treatment (Figure 5B). It is clear from the experiment that a cocktail of proteins P16-17/100 and BP404 was efficient in disrupting a dual biofilm formed by *S. aureus* Rumba and E. faecalis. In addition to disruption of the biofilm, the cocktail was also efficient in causing significant damage to the bacterial cells embedded in the biofilm.

## 3. Discussion

In this work, we evaluated the potential of two chimeric enzybiotics in disrupting a dual biofilm formed by *S. aureus* Rumba, a veterinary isolate, and E. faecalis, a clinical isolate. One of the chimeric enzybiotics used in this study, BP404, was earlier found to be active against *E. faecalis* ATCC 29,212 and *E. faecalis* clinical isolate [34], with a higher activity exhibited against *E. faecalis* clinical isolate. However, the study was performed against planktonic cells of E. faecalis. We wanted to test the activity of this protein against the biofilm formed by *E. faecalis* clinical isolate on an inert surface. In nature, the existence of biofilms is often polymicrobial, which comprises of different phyla or species which interact with each other in the complex community [6]. The formation of biofilms is significant in chronic wound infections in people with diabetic foot ulcers. For instance, in a study where the wound tissue biopsies were analyzed by electron microscopy, biofilms were present in 60% of chronic wounds compared to only 6% for acute wounds [38]. Alarmingly, biofilm-infected foot ulceration in diabetic foot ulcers contributes to more than 80% of lower-limb amputations [39], consequently causing increased chances of death within 18 months [40]. It has also been reported that the most appropriate techniques for revealing biofilm formation in biopsies include confocal microscopy, fluorescence in situ hybridization and scanning electron microscopy [41].

*Staphylococcus aureus* is the most common pathogen present in the biofilms formed in diabetic foot ulcers. A polymixed biofilm comprised of *S. aureus*, *E. faecalis*, *P. aeruginosa*, and *A. baumannii* is very commonly associated with diabetic foot ulcers [42]. Interestingly, it has been reported that these polymixed communities constitute a thicker biofilm in comparison with monobacterial biofilms, and were difficult to eradicate [43]. This observation was corroborated by our result in this study, in which a thicker biofilm was formed with *S. aureus* Rumba and *E. faecalis* clinical isolate as compared to mono species biofilm formation, as estimated by crystal violet staining. 

Alleviation of biofilms and annihilation of embedded bacterial cells is the most important aspect in the treatment of biofilm-associated infections. Most of the antibiotics are impotent against bacterial biofilms, especially since the EPS of the biofilm restricts entry of antibiotics to the embedded bacteria [42]. Biofilms have been found to be 10–1000 times more resistant to antibiotics as compared to the planktonic style of growth. In a work by Mottola et al. (2016) [44], only ceftaroline and gentamicin were found to eradicate *S. aureus* biofilms. Although physical treatment or debridement performed by surgical instruments partially remove bacterial biofilms in infected tissues, the disadvantage is that debridement should be regularly repeated at specific intervals to completely disrupt biofilms [45,46]. However, it is much more difficult to remove biofilm formed on medical devices and surgical implants, as the biofilms are highly resistant to heavy metals and ultraviolet exposure [2]. 

Bacteriophages have been found to be effective in removing biofilms, by penetrating the existing biofilm mesh and eliminating the embedded bacteria [47,48]. Phages may also possess depolymerases that facilitate biofilm disruption. In addition, phage-derived endolysins are also efficient mediators of biofilm removal [49,50,51]. The rapid emergence of bacterial resistance to phages by Crispr or similar mechanisms limits the use of intact phages against planktonic- and biofilm-embedded bacteria. However, phage-derived proteins against which bacteria are unable to develop resistance have emerged in the last decade as an alternative to antibiotics to treat drug-resistant bacteria following a planktonic- or biofilm-mediated lifestyle. Nevertheless, while the mechanism of disruption of biofilm by lysins and enzybiotics is not fully understood, it has been shown in several studies that endolysins could degrade a large amount of the EPS of biofilm [52]. 

In our study, a cocktail of two chimeric proteins has been found to be very active in disrupting the dual species biofilm formed by *S. aureus* Rumba and *E. faecalis* clinical isolate. In addition to the disruption of biofilms, the chimeric proteins also caused the death of biofilm-embedded bacteria amounting to more than 90% within 3 h of treatment. The individual proteins (BP404 or P16-17/100) could also exhibit biofilm disruption and mortality of bacteria in the biofilm, albeit at a lower efficiency as compared to the cocktail. It can be speculated that the annihilation of biofilm-encased bacteria may cause a structural change in the biofilm mesh, hence contributing to the disruption of the biofilm. Importantly, a longer period of incubation with individual proteins was needed to achieve the same effectiveness as that of the cocktail-treated biofilm. 

The cocktail used in this study is comprised of proteins, BP404 and P16-17/100. BP404 is comprised of the N-terminal catalytic domain and C-terminal cell-wall binding domain from PlyV12, which targets E. faecalis. In contrast, the P16-17/100 had a *S. aureus*-specific cell-wall-binding domain from protein P17 from phage ϕ44AHJD. However, in our study, we noticed that both these proteins could also bind to the alternate bacterial strain. This cell-wall binding has also contributed to the activity of both the proteins against both the bacterial strains. This is one of the prime reasons why the cocktail was efficient against the dual biofilm.

This is the first instance where a cocktail of chimeric enzybiotics has been tested against a dual biofilm under in vitro conditions. The use and activity of these proteins are particularly interesting as the dual biofilm was formed by *S. aureus* and *E. faecalis*. These pathogenic bacteria are commonly associated with diabetic foot ulcers, and form difficult-to-treat biofilms in the wounds. An in vivo study is further warranted to depict clearly the efficiency of these proteins under natural conditions.

## 4. Materials and Methods

### 4.1. Bacterial Strains and Growth Conditions

The *E. faecalis* clinical isolate and *S. aureus* Rumba were used to evaluate the antibiofilm and antibacterial efficiency of the protein. *E. faecalis* was collected from Buraidah Central Hospital, Buraydah, Saudi Arabia. *S. aureus* Rumba was a veterinary isolate from the udder of a cow with bovine mastitis. *S. aureus* Rumba was a gift from the Prof. Dr. Udo Blaesi lab, Max F. Perutz Laboratories, Vienna, Austria.

Luria Bertani (LB) media/agar (1% NaCl, 0.5% yeast extract, 1% peptone, 1.5% Agar pH: 7.0, Micromaster, India) was used for culturing of the bacterial strains. Unless otherwise mentioned, all the bacterial strains were grown under shaking conditions (150 rpm) at 37 °C. The bacterial strains were maintained as glycerol stock (50% *v*/*v*) at −80 °C for long-time storage. The purity of the bacterial strains was checked by streak plating onto LB agar plates before inoculation.

### 4.2. Software Used for Protein Structure Prediction

The Phyre program [35] was used to predict the three-dimensional structure of the protein. The prediction was made at a confidence level of 100% with a sequence coverage of 44%.

### 4.3. Extraction of Phage DNA

Extraction of the phage DNA was done with modifications of the protocol described by Jakočiūnė et al. [53]. In short, phage ϕ44AHJD was mixed with *S. aureus* Rumba bacteria, and top agar (0.75% agar) was added. The mixture was swirled shortly and was poured onto pre-made LB agar plates. The plates were incubated at 37 °C for 18 h to achieve a confluent lysis of *S. aureus* Rumba cells. The top layer of the *S. aureus* Rumba-lysed LB plates was eluted out by incubating at 4 °C overnight after the addition of 5 mL of SM buffer (0.2% MgSO_4_∙6H_2_O, 0.58% NaCl, 5 mL/100 mL of 1 M Tris-Cl pH 7.5). The supernatant was filter-sterilized over a 0.22 µM filter after centrifugation of the elute at 6000 rpm for 10 min. The filtered elute (500 µL–1.0 mL) was treated with 1 unit of DNase I (Fermentas, Waltham, MA, USA) and 10 µg of RNase A (Fermentas, Waltham, MA, USA) to remove the host DNA and RNA. The DNase I and RNase A treatment was allowed to proceed for 2 h at 37 °C, after which 20 µL of 0.5 M EDTA (Fluka, Muskegon, MI, USA) was added to stop the reaction. To digest the phage capsid, 1.25 µg of the enzyme Proteinase K was added and incubated for 2 h at 56 °C. The phage nucleic acid was precipitated with the addition of 2× 99% ethanol at −70 °C for 18 h. After centrifugation at 13,000 rpm for 10 min, the phage DNA pellet was washed with 70% ethanol. The pellet was air-dried and resuspended in autoclaved distilled water. The DNA was checked by resolving onto a 1% agarose gel. The DNA was used for PCR amplification of the desired genes.

### 4.4. PCR Amplification of Genes

The gene encoding for the catalytic domain (CD) of protein P16 (423 bp) (GenBank: NC_004678.1) [54] and the cell-wall-binding domain (CWD) from P17 (300 bp) (GenBank: NC_004678.1) [54] was PCR-amplified with primers P16CDFP and P16CDRP for P16 and P17CWDFP and P17CWDRP for P17 (Table 1). The PCR primers were manually designed and the Tm for each primer pair was evaluated by the Gene Runner program. The PCR reaction set up for gene amplification with ϕ44AHJD phage DNA as a template is as follows: 94 °C for 8 min, 94 °C for 60 s, 63 °C for 25 s, 72 °C for 90 s, and 72 °C for 7 min. The reaction was set up to continue for 35 cycles; 2× Pfu master mix (G-Biosciences, St. Louis, MI, USA) was used for setting up of the reaction. The expected PCR product was confirmed for its size (423 bp and 300 bp) by resolving on a 0.9% agarose gel.

### 4.5. Cloning of Gene P16CD and P17CWD

The 423 bp gene fragment (P16CD) amplified with the primer set P16CDFP and P16CDRP was purified from the gel by a GeneJET Gel extraction kit (Thermo Fisher Scientific, Waltham, MA, USA). Similarly, the 300 bp cell-wall-binding domain (P17CWD) encoding gene fragment amplified by the primers P17CWDFP and P17CWDRP was also purified out of the gel by a GeneJET Gel extraction kit. The *P16CD* gene fragment was digested with BamHI/KpnI (Thermo Fisher Scientific, Waltham, MA, USA) and cloned into a restriction site, BamHI/KpnI, of the protein expression vector pQE30, creating the plasmid pQE-P16 (Table 2). The purified *P17CWD* was restricted with KpnI/HindIII. The restricted P17CWD gene was cloned into downstream of the P16CD in the plasmid pQE-16, creating the new plasmid pQE-P16-17/100. The cloning was confirmed by PCR amplification and restriction analysis. The sequence of the cloned gene was confirmed by gene sequencing.

### 4.6. Synthesis and Purification of Protein P16-17/100

The pQE-P16-17/100 plasmid was induced with 0.1 mM of IPTG after growing the *E. coli* BL21(DE3) cells to an OD_600_ of 0.4–0.5. Protein synthesis was facilitated for 16 h at 17 °C following IPTG induction of the cultures. A small aliquot of the uninduced and induced cultures was resolved on a 12% SDS-PAGE gel to verify the synthesis of protein P16-17/100. The induced culture was pelleted by centrifugation at 6000 rpm for 50 min. The cell pellet was stored until further use at −80 °C. 

Since the protein was expressed in inclusion bodies, denaturation purification was used to solubilize and purify the protein with minor modifications from the standard protocol described by Qiagen (St. Louis, MO, USA). Briefly, 10 mL of the denaturation lysis buffer B (100 mM NaH_2_PO_4_, 10 mM Tris-Cl, 8 M Urea, pH 6.5) was used to resuspend the induced cell pellet. The resuspended pellet was sonicated with pulse sonication cycles (50% power, 4 s pulse, 5 s off; Biosafer, Zhichunli, Beijing, China) for 10 min in ice. The mixture was incubated at 20 °C, shaking for 16 h for complete solubilization of the protein. The supernatant was collected after centrifugation for 45 min at 16 °C. The urea solubilized protein was bound to a Ni-NTA agarose column (G-Biosciences, St. Louis, MO, USA) by incubating the calibrated Ni-NTA agarose beads (1.0 mL) to the supernatant for 45 min at room temperature. The protein-bound Ni-NTA was loaded onto a column, and the supernatant was drained by gravity flow. The protein-bound Ni-NTA beads were washed thrice with 10 times bed volume of buffer C (100 mM NaH_2_PO_4_, 10 mM Tris-Cl, 20 mM Imidazole, 8 M Urea pH 6.5). The proteins were eluted out as 1.0 mL fractions from Ni-NTA using buffer D (100 mM NaH_2_PO_4_, 10 mM Tris-Cl, 250 mM Imidazole, 8 M Urea pH 6.5). Purity levels of the eluted proteins were estimated by resolving on a 12% agarose gel. The concentration of the proteins was estimated by nanodrop (Bio-Rad, Des Plaines, IL, USA). Stepwise dialysis was employed for refolding of the protein with the urea concentration reducing from 8 M to 0 M. The final dialysis was made in buffer Z (20 mM Tris, 300 mM NaCl, 20 mM MgCl_2_, pH: 6.5). Activity of the protein was assessed by live–dead staining of bacteria followed by treatment with proteins.

### 4.7. Biofilm Formation and Analysis of the Microbial Biomass

The ability of both the bacterial strains *S. aureus* Rumba and *E. faecalis* clinical isolate to form biofilm on a glass surface was evaluated by immersing a glass coverslip in a culture of the bacterial strains grown on LB media. Initially, 1.0 mL of overnight grown culture (approx. 1 × 10^8^ CFU/mL) was inoculated to 15 mL of LB and incubated for 24 h at 37 °C. Sterile glass coverslips were immersed onto the inoculated bacterial culture. The bacteria-grown LB media was replaced with fresh LB media at every 24 h. Biofilm formation and the intensity of the microbial biomass was estimated by confocal microscopy and by crystal violet staining at every 24 h until 120 h. For microscopy analysis and crystal staining, the immersed glass coverslip was taken at the end of every 24 h, and unattached planktonic bacterial cells were washed away thrice with distilled water. Live/dead staining of the cells in the biofilm formed on the glass coverslips was evaluated by staining with a Syto9/Propidium iodide mix (Thermo Fisher Scientific, Waltham, MA, USA: 5 µM/500 nM) for 10 min at room temperature. Imaging of the live and dead cells (SYTO9 excitation/emission at 483/503 nm: propidium iodide excitation/emission at 535/617 nm) was done with a spinning-disk confocal microscope (Zeiss, Jena, Germany). A Rolera Em-C^2^ camera was used for image capturing. The objective used for imaging was 63 × oil immersion (Zeiss, Jena, Germany). The captured images were processed with the Zen lite software (Zeiss, Jena, Germany). The number of live (green) and dead (red) cells per frame/picture was counted manually. The experiment was performed in triplicate, and the mean value was taken for the calculations. The crystal violet staining of the microbial biomass was done according to the protocol described by Lemos et al. [55]. In short, after washing of the glass coverslips with distilled water, 500 μL of 0.1% crystal violet (Avonchem, Macclesfield, UK) in distilled water was added to the slides. The staining was allowed to continue for 15 min. The slides were further washed thrice to remove any residual stain. One mL of 33% acetic acid was added to the coverslip and incubated for 10 min at room temperature. The stain released after addition of acetic acid was transferred to a cuvette, and the absorbance was measured at an optical density of 575 nm.

### 4.8. Cell-Wall-Binding of the Protein towards Bacterial Cells

The binding efficiency of the recombinant protein towards *S. aureus* Rumba and *E. faecalis* clinical isolate was analyzed by adding 60 ng of the protein to 1 × 10^7^ CFU of logarithmically growing bacterial cells in buffer z, pH 6.5, followed by incubation at 37 °C for 20 min. The supernatant was extracted after centrifugation of the mixture at 13,000 rpm for 5 min following incubation. The unbound protein in the supernatant fraction was precipitated with TCA. In order to exclude the possibility of nonspecific binding of the protein, the cell pellet was washed thrice with 1× PBS buffer. SDS-PAGE gel loading dye was added to the pellet and the sample was boiled for 10 min at 95 °C. After resolving both the supernatant-precipitated and cell-pellet-bound proteins on an SDS-PAGE gel, Western blotting was performed. For Western blot analysis, the gel-resolved proteins were transferred to nitrocellulose membrane by a semidry Western blotting apparatus (Bio-rad, Hercules, CA, USA) for 20 min at 20 V. The blot was blocked with 5% milk powder in 1 × TBST buffer for 1 h at room temperature under shaking conditions (50 rpm). Following washing of the membrane with 1 × TBST buffer, primary antibody (mouse anti-his antibody, Abclonal, Woburn, MA, USA) was added to the blot in a dilution of 1:2000 (stock concentration: 0.2 mg/mL; diluted concentration used: 80 ng/mL) and was incubated for 16 h at 20 °C under shaking conditions (50 rpm). Secondary antibody (goat anti-mouse IgG linked to alkaline phosphatase, Elabscience, Houston, TX, USA) was further added to the blot in a dilution of 1:12,000 (stock concentration: 0.2 mg/mL; working concentration: 16.66 ng/mL) after washing of the blot with 1 × TBST buffer. The blot was incubated at room temperature for 1 h under shaking conditions (50 rpm). After further washing, the blot was developed by the addition of 5 mL of BCIP/NBT (G-Biosciences, St. Louis, MI, USA) substrate. 

### 4.9. Software Used for Graph Preparation Statistical Analysis of the Data

The *p*-values and statistical variation of data were calculated using a one-way ANOVA calculator (https://goodcalculators.com/one-way-anova-calculator/ (accessed on 20 March 2023)). The acquired data were graphically represented using Microsoft Excel (2010 version). The mean values and standard deviation for the dataset were calculated with Microsoft Excel (2010 version). Error bars in the graph represent the standard deviation.

## 5. Conclusions

Enzybiotics are well studied for the elimination of drug-resistant planktonic bacteria and its biofilms. Our studies show that a cocktail of chimeric enzybiotics is efficient in reducing preformed dual biofilm formed on an inert surface. Apart from biofilm disruption, the enzybiotics are also efficient in killing biofilm-embedded bacterial cells. This work states that a cocktail of enzybiotics or recombinant proteins can be designed as an effective tool in disrupting and removing biofilm from inert surfaces and presumably from wound infections, particularly of diabetic foot ulcers. 

## Figures and Tables

**Table 1:** Primers used for the study.

PCR Primers	Sequence	Notes
P16CDFP	5′ACCAAGGGATCCATGAAATCACAACAACAAGC3′	Forward primer for P16 CD gene amplification
P16CDRP	5′CATAGGTACCATTACTACCTGAAAATTTAGGTCT3′	Reverse primer for P16 CD gene amplification
P17CWDFP	5′CATAGGTACCATCAAAACTGACGCACCATAT3′	Forward primer for P17 CWD gene amplification
P17CWDRP	5′CAGGAAGCTTCTATTTTTGATGTTTTGCTACC3′	Reverse primer for P17 CWD gene amplification

**Table 2:** Plasmids constructed for this study.

Plasmid Name	Notes	Cloning Site
pQE-P16	423 bp gene from P16 protein encoding gene; cloned under lasUV5 promoter in pQE30 vector	BamHI/KpnI
pQE-P16-17/100	300 bp gene from P17 protein encoding gene; cloned downstream of P16 in pQE-P16 vector	KpnI/HindIII

## Data Availability

Data is contained within the article.
